# Effects of *Nostoc commune* extract on the cerebral oxidative and neuroinflammatory status in a mice model of schizophrenia

**DOI:** 10.1016/j.bbrep.2023.101594

**Published:** 2023-12-07

**Authors:** Parisa Jahani Bahnamiri, Akbar Hajizadeh Moghaddam, Mojtaba Ranjbar, Ehsan Nazifi

**Affiliations:** a Department of Biology, Faculty of Basic Sciences, University of Mazandaran, Babolsar, Iran; b Faculty of Biotechnology, Amol University of Special Modern Technologies, Amol, Iran; c Department of Plant Sciences, University of Mazandaran, Babolsar, Iran

**Keywords:** *Nostoc commune*, Ketamine, Schizophrenia, Oxidative damages, Inflammatory cytokines, Mice

## Abstract

Cyanobacterium *Nostoc commune* has long been used to alleviate various diseases. This research examines the effects of *Nostoc commune* extract (NCE) against behavioral disorders, cerebral oxidative stress, and inflammatory damage in the ketamine-induced schizophrenia model. Oral NCE administration (70 and 150 mg/kg/d) is performed after intraperitoneal ketamine injection (20 mg/kg) for 14 consecutive days. The forced swimming and open field tests are used to assess schizophrenia-like behaviors. After the behavioral test, dopamine (DA) level, oxidative stress markers, as well as the interleukin-6 (IL-6) and tumor necrosis factor-α (TNF-α) expression are measured in the cerebral cortex. The results show that NCE treatment ameliorates KET-induced anxiety and depressive-like behaviors in OFT and FST, respectively. NCE considerably decreases the malondialdehyde (MDA) and DA levels and IL-6 and TNF-α expressions in mice with schizophrenia-like symptoms. Also, a significant increase is observed in the glutathione (GSH) level and catalase (CAT), superoxide dismutase (SOD), and glutathione reductase (GRx) activity in cerebral tissue. The present study shows that NCE treatment effectively improves KET-induced schizophrenia-like behaviors and oxidative and inflammatory damage. Therefore, NCE, via its bioactive constituents, could have strong neuroprotective effects in the schizophrenia-like model.

## Highlights

• *Nostoc commune* extract improve neuroinflammation in SCZ-like mice.
• *Nostoc commune* extract attenuates depressive and anxiety-like behavior.
• *Nostoc commune* extract is useful for oxidative stress injury in SCZ-like mice.

## Graphical abstract

**Figure:**
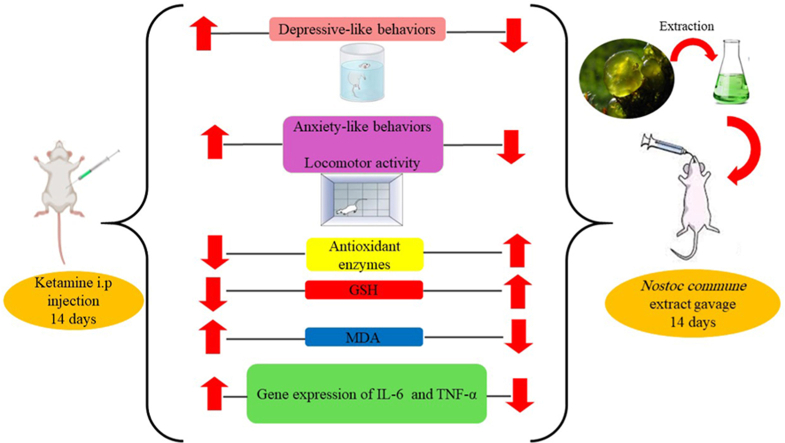

## 1 Introduction

Schizophrenia (SCZ) is a chronic, devastating psychiatric disorder with complex alterations in neuroimmune pathways [1]. SCZ patients display various behavioral disturbances, including negative symptoms (poor social functioning, amotivation, depressed mood), positive symptoms (delusions, hallucinations), and memory dysfunction [2,3]. Although dysregulation of dopaminergic neurotransmission plays a significant role in SCZ [4], previous studies have suggested the importance of oxidative stress [5] and inflammatory mechanisms in SCZ pathogenesis [6]. The antioxidant system impairment or elevated reactive oxygen species (ROS) leads to biological macromolecule destruction, lipid peroxidation progression, and gene expression alteration [7]. In addition, immunological responses such as enhanced inflammatory biomarkers, including TNF-α and IL-6, by activated microglia cause brain damage and disease progression in SCZ patients [6]. IL-6 has been reported in the kynurenine up-regulating, an endogenous antagonist of *N*-methyl-d-aspartate receptors (NMDAR), which indicates impaired neurotransmission in response to chronic neuroinflammation in SCZ [8,9]. In psychopathology studies, the pharmacological model of ketamine (KET), a non-competitive antagonist of NMDAR, can induce psychosis in rodents and humans [10]. KET is a general anesthesia drug whose neurotoxicity effects depend on consumption dose and time [11]. Chronically used KET at subanesthetic doses induces NMDAR dysfunction, elevated intracellular calcium influx, and thus redox dysregulation. NMDAR dysfunction could be responsible for abnormal downstream dopamine (DA) activity by inhibiting the γ-aminobutyric acidergic (GABA) system. Thus, KET administration can induce negative symptoms, delusions, and paranoia similar to SCZ symptoms [12]. Also, evidence related to Anti-NMDAR encephalitis has approved clearly that NMDAR hypofunction can result in psychosis and other mood changes of SCZ [13].

Because of the antipsychotic medication’s adverse effects and poor efficacy in behavior deficit amelioration, SCZ treatment remains challenging [14]. Today, natural compounds are used broadly to prevent and treat neuropsychological diseases [15]. *Nostoc commune* is an edible blue-green alga of the Nostocaceae family that often forms visual colonies in freshwater and terrestrial systems such as rice paddies [16]. It contains a broad spectrum of bioactive components, including amino acids, minerals, vitamins, fatty acids, polysaccharides, and UV-absorbing pigments, applied for pharmacological uses [17,18]. Due to its unique compounds*, Nostoc commune* extract (NCE) has several biological activities, such as antibacterial [19], antioxidant [20], immunomodulation [21], and antifungal properties [22]. The significant role of polysaccharides and UV-absorbing pigments (mycosporine-like amino acids and scytonemin) is well-proven in the antioxidant and anti-inflammation effects [[19], [20], [21]]. Studies indicate this alga effectively improves inflammation, heart and liver diseases, anxiety, and chronic fatigue [18,23]. Also, *in vivo* and in vitro investigations indicate that NCE is an effective supplementation for wound healing [24], colon colitis [25], obesity dysfunction [26], serum cholesterol-lowering [18], and tumorigenesis inhibition [27]. Despite the widespread use of *Nostoc commune* as a therapeutic agent in diseases, its neuroprotective properties have not been investigated. Accordingly, this research is designed to examine the neuroprotective impacts of NCE by evaluating behavioral disorders, oxidative stress, and neuroinflammation biomarkers in a mice model of SCZ.

## 2 Method & materials

### 2.1 *Nostoc commune* collection and extraction

Colonies of the *Nostoc commune* after the rain were collected in October 2020 from the Shirgah, Mazandaran province, Iran (36°11′14.7″N 52°57′30.1″ E). Wet colonies were washed to remove soil and air-dried for 2 weeks in the laboratory. The *Nostoc commune* powder (150 g) was macerated in 80% methanol (1500 mL) for 24 h and then filtered with double paper filters. This process was repeated three times. The filtered extract was concentrated using a rotary evaporator under decreased pressure at 39 °C. The obtained semi-solid residue was dried in the oven incubator for three days [26]. Voucher specimens were deposited in the herbarium of Mazandaran University (HUMZ, 9136) according to standard herbarium methods.

### 2.2 Animals

All study procedures were done according to the care guidelines followed by the National Institutes of Health guide for the care and use of Laboratory animals (NIH Publications No. 8023, revised 1978); the ethics committee of the University of Mazandaran, Babolsar (IR.UMZ.REC.1399.015), approved the maintenance and use of animals. Thirty-five adult male mice weighing 20–25 g were obtained from the Pasteur Institute, Amol, Iran. They were kept in 5 cages at 22 ± 1 °C under a 12/12 h light/dark cycle with 65% ± 5% humidity. The animals were fed water and a standard rodent pellet diet ad libitum. They were accustomed to the laboratory conditions for at least one week. All behavioral tests were performed between 8:00 a.m. and 2:00 p.m.

### 2.3 Experimental design

**Fig. 1:**
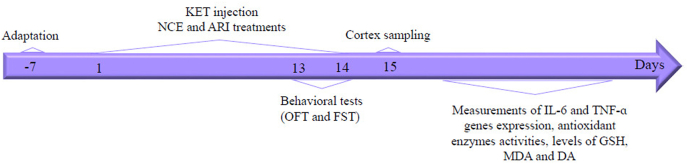
Timeline of the experimental design.

Fig. 1 depicts an overview of the experimental design. This investigation randomly assigned animals to 6 groups (7 mice per group). The control group was treated with 0.9% saline, while the NCE150 group was treated with NCE (150 mg/kg). The SCZ group was injected with KET (20 mg/kg, i. p) for 14 days. The ARI group was treated with aripiprazole (10 mg/kg orally), and the NCE groups were treated with NCE (70 and 150 mg/kg orally) following KET injection for 14 days. Then, the behavioral tests were applied to all animals before their sacrifice. Eventually, the brain cortex samples of mice were isolated for biochemical and gene expression assays.

### 2.4 Behavior tests

#### 2.4.1 Open-field test (OFT)

This test was performed to assay locomotor activity and anxiety-like behaviors in rodents. Each mouse was placed in a black box (100 × 100 × 40 cm), and the floor of the apparatus was divided into 25 equal quadrants. For each mouse, parameters of the number of crossing lines and the time spent in the central apparatus zone for 10 min were used [28].

#### 2.4.2 Forced swimming test (FST)

FST was used to assess depressive-like behaviors according to the Dalvi and Lucki method. Briefly, mice were located in a cylinder (25 cm diameter and 40 cm height) filled with fresh water (22–26 °C). Then, forced swimming was performed for 6 min, and the time of swimming and immobility for 5 min was recorded [29].

### 2.5 Preparation of tissue homogenate

The cortex tissue (150 mg) was homogenized in 1 mL of buffer (10 nmol/L Tris-HCl, pH = 7.4, 1 mmol/Ll EDTA, & 0.32 mol/L sucrose); it was then centrifuged (13,600 g, 4 °C, 30 min). The supernatant was utilized for biochemical evaluations.

### 2.6 DA level measurement

The DA level in the brain was assessed based on the method introduced by Guo et al. For the calculation of cerebral DA level, the cortex supernatant (1 mL) was added to 1 mL potassium ferricyanide (1.5 × 10^−2^ M) and 1 mL ferric chloride (1.5 × 10^−2^ M). The samples were diluted to 25 mL with distilled water; and then kept at room temperature for half an hour. The absorbance was measured at 735 nm, and the results were defined as ng/mg protein [30].

### 2.7 Determination of antioxidant enzymes activity

The superoxide dismutase (SOD) and catalase (CAT) activity were measured based on the Genet method. The SOD absorbance was examined at 420 nm and 25 °C for 2 min; further, its activity was well-defined as the amount of an enzyme causing 50% half-maximally autoxidation of pyrogallol inhibition. The CAT activity was presented as μmole of H_2_O_2_ consume/min/mg protein, and its enzyme absorbance was measured at 240 nm and 25 °C for 120s [31]. The glutathione reductase (GRx) activity was measured by the Pinto method. Enzyme absorbance was measured at 340 nm, and one GRx unit was considered 1 μmol of NADPH oxidized/min/mg protein [32].

### 2.8 Glutathione (GSH) level measurement

The GSH level was measured according to Fukazawa & Tokumura method [30]. In summary, 200 μl of cortex homogenates were mixed with 130 μl of DTNB (0.04%) and 1.1 mM of sodium phosphate buffer (0.25 M, PH 7.4). Distilled water was added to a final volume of 1.5 mL, and GHS absorbance was presented as mg GSH/gr protein at 412 nm [33].

### 2.9 Malondialdehyde (MDA) level measurement

MDA is a biological marker of Lipid peroxidation. In this study, its level was measured by the Esterbauer & Cheeseman method. In brief, 200 L of supernatant was combined with a reaction mixture including 1 mL of thiobarbituric acid (0.67%) and 0.5 mL of trichloroacetic acid (20%). Next, this combination was incubated in a boiling water bath (100 °C, 1 h) and was centrifuged after cooling. MDA absorbance was expressed as μg/mg protein at 535 nm [34].

### 2.10 Protein content measurement

According to the Bradford method, the protein of homogenate cortex samples was measured using bovine serum albumin (BSA) as standard [35].

### 2.11 IL-6 and TNF-α estimation

The gene expression levels of IL-6, TNF-ɑ, and GAPDH were assayed using real-time PCR. All stages were executed according to the manufacturer’s instructions: 1) employing an RNeasy Mini Kit, total RNA was extracted from the hippocampus; 2)The RNA concentration was assayed spectrophotometrically at 260 and 280 nm,3) 1 μg of total RNA was treated with RNase-free DNase I (Fermentas) to removing genomic DNA, 4) synthesis of first-strand cDNA was done with a Superscript III Reverse Transcriptase (Fermentas) and the sequences of the primer used for qRT-PCR represent in Table 1, 5) The cDNA was assessed with the SYBR Green kit (TaKaRa) by Real-Time PCR and thermal cycling was performed using Corbett Rotor-Gene 6000 system. Finally, Gene expression was compared according to the 2^−ΔΔCT^ method and Ct values [36].

**Table 1:** Sequences of primers used in qRT-PCR.

Gene	Primer	Sequence	Amplicon lengths (bp)
GAPDH	forward	5′-ATCCTGCACCACCAACTGC-3′	129
	reverse	5′-ACGCCACAGCTTTCCAGAG-3′	
TNF-α	forward	5′-GGAGGAGCAGCTGGAGTG-3′	131
	reverse	5′-CCTTGAAGAGAACCTGGGAGTAGA-3′	
IL-6	forward	5′-TCACAGAGGATACCACCCACAA-3′	146
	reverse	5′-CAGTGCATCATCGCTGTTCATAC-3′	

### 2.12 Data analyses

GraphPad Prism (V 8.0.2, GraphPad Software, Inc) was used for data analyses. All data were presented as Mean ± S. D utilizing one-way ANOVA followed by Tukey’s test; P < 0.05 was considered statistically significant.

## 3 Results

### 3.1 UV absorption spectra of NCE

**Fig. 2:**
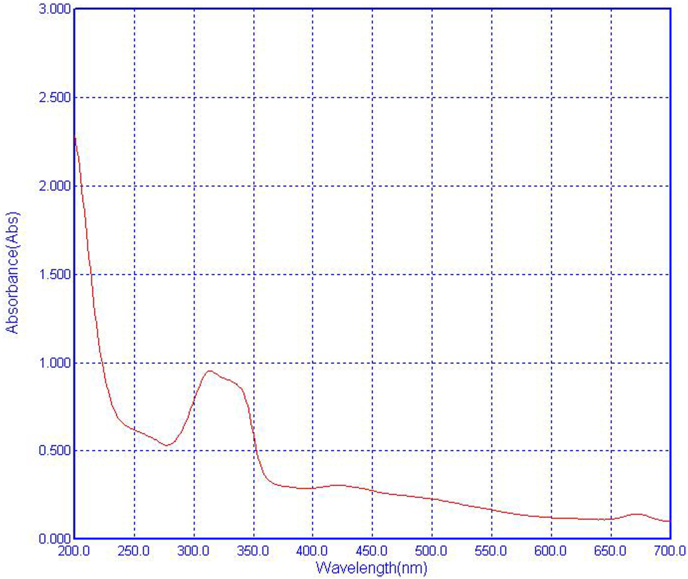
UV absorption spectra of NCE soluble in distilled water.

Fig. 2 shows the UV absorption spectrum of NCE soluble in distilled water. The absorption spectrum of NCE was similar to that obtained from *Nostoc commune* (genotype B) in the previous study by Nazifi et al. [20]. The absorption spectra of both extracts showed the absorption maxima at 312 and 340 nm. Nazifi et al. demonstrated by the High-performance liquid chromatography method (HPLC) that this characteristic absorption maxima are related to the presence of mycosporine-like-amino acids (MAAs) with molecular weights of 880 Da, 273 Da, and 1050 Da. The main MAA was the 1050-Da MAA with a characteristic absorption maxima at 312 and 340 nm, which consisted of two cyclohexenones and one cyclohexenimine ring. As previously reported, MAAs are potent antioxidant and anti-inflammatory compounds with pharmaceutical applications [20,22].

### 3.2 NCE treatment effects on anxiety-like behaviors

**Fig. 3:**
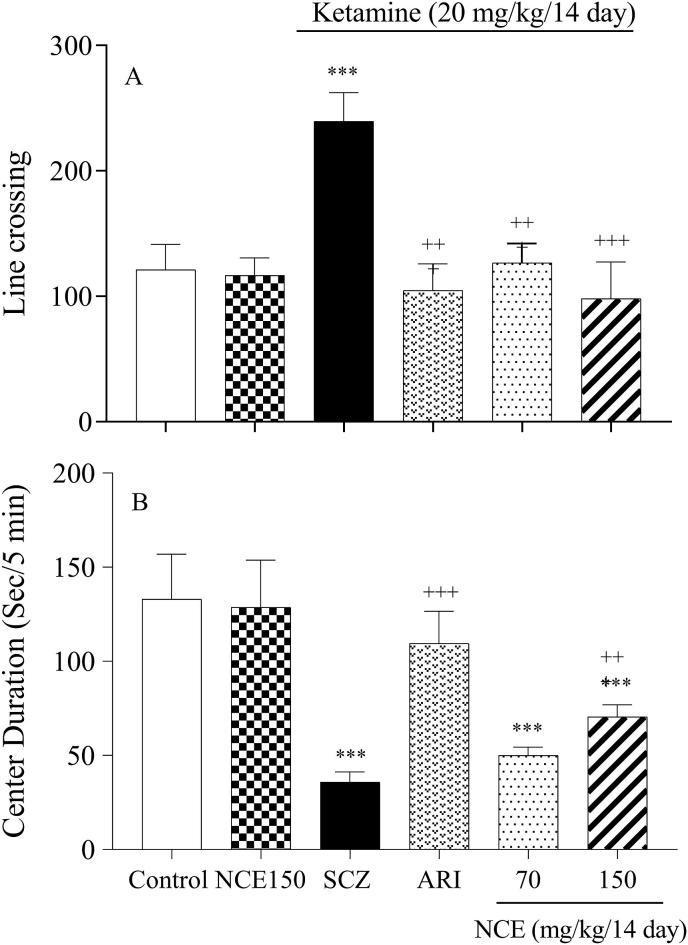
NCE treatment effects on locomotor activity and anxiety-like behaviors in KET-induced SCZ. Values were expressed as means ± SD for 7 mice. SCZ; schizophrenia, ARI; aripiprazole, NCE (*Nostoc commune* extract) 70 and 150 mg/kg ***P < 0.001 as compared to control group. +++ P < 0.001 as compared to the SCZ group.

In the open field test, locomotor activity was significantly increased in the SCZ group as compared to the control group (P < 0.001), and treatment with NCE (70 and 150 mg/kg) significantly decreased line crossing and locomotor activity as compared to the SCZ group (P < 0.001). On the other hand, KET injection led to a significant decrease in time spent in the center for SCZ mice compared to the control group (P < 0.001). NCE treatment (150 mg/kg) reversed anxiogenic-like behaviors induced by KET (P < 0.001) as compared to the SCZ mice (Fig. 3A and B).

### 3.3 NCE treatment effects on depressive-like behaviors

**Fig. 4:**
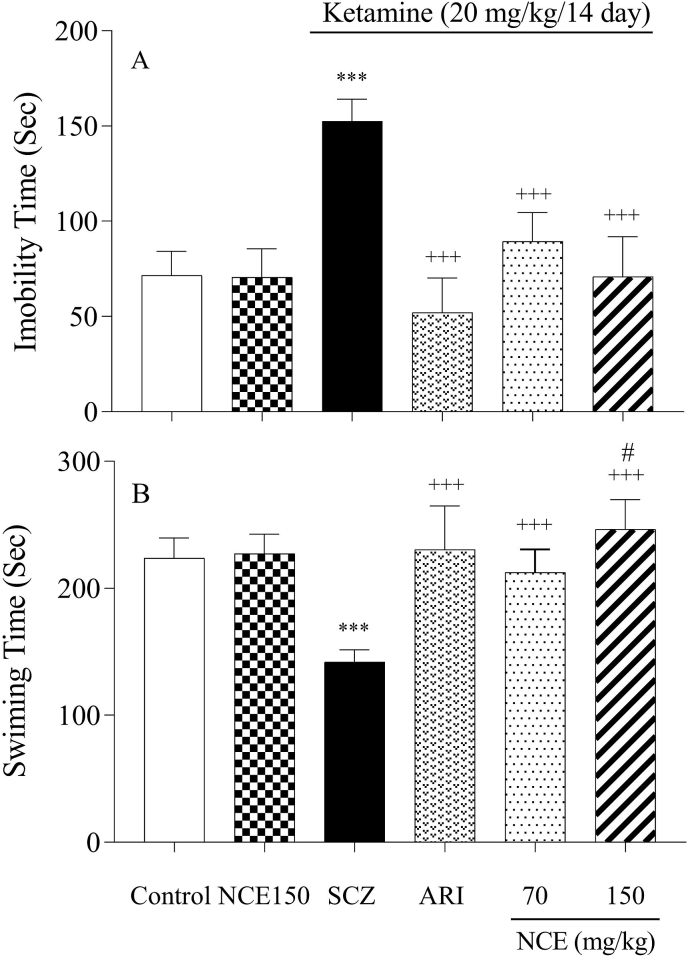
NCE treatment effects on depressive-like behaviors in KET-induced SCZ. Values were expressed as means ± SD for 7 mice. SCZ; schizophrenia, ARI; aripiprazole, NCE (*Nostoc commune* extract) 70 and 150 mg/kg ***P < 0.001, as compared to the control group. +++P < 0.001 as compared to the SCZ group, #P < 0.05 compared to 70 mg/kg of NCE.

Fig. 4A and B depict the depressive-like behaviors in FST. One-way ANOVA analyses show that SCZ induction notably elevates immobility time and reduces swimming time in FST compared to the control group (P < 0.001). NCE treatments significantly reduced immobility time and led to increasing swimming time compared with the SCZ group (P < 0.001). Also, the dose of 150 mg/kg significantly increased compared to NCE at the dose of 70 mg/kg (p < 0.05).

### 3.4 NCE treatment effects on DA levels

**Fig. 5:**
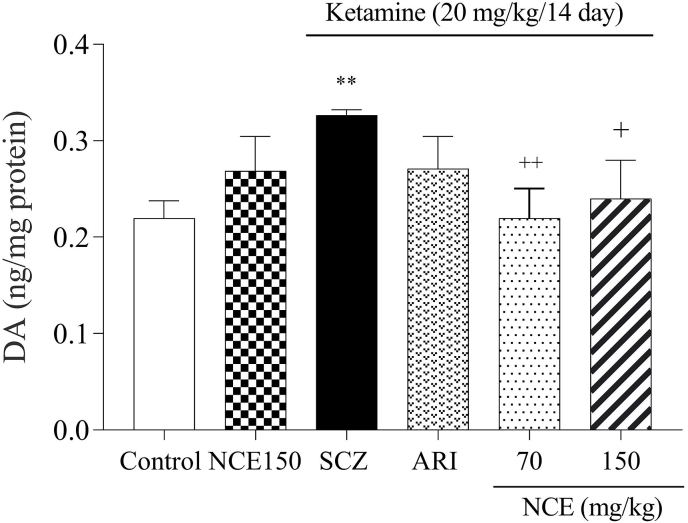
NCE treatment effects on DA level in KET-induced SCZ. Values were expressed as means ± SD for 7 mice. SCZ; schizophrenia, ARI; aripiprazole, NCE (*Nostoc commune* extract) 70 and 150 mg/kg). **P < 0.01, as compared to the control group. +P < 0.05, ++P < 0.01 as compared to the SCZ group.

In the current study, one-way ANOVA results revealed that the KET-induced SCZ group exhibited a significant increase in DA levels compared to the control group (p < 0.01). Meanwhile, the NC extract treatment at the dose of 70 and 150 mg/kg significantly decreased the DA levels compared to the SCZ group (p < 0.01, p < 0.05), respectively (Fig. 5).

### 3.5 NCE treatment effects on antioxidant enzyme activities

Biochemical analyses in Table 2 showed that the SOD, CAT, and GRx activities were significantly reduced in the SCZ group compared to the control group (P < 0.001). NCE treatment (70 mg/kg) did not improve antioxidant enzyme activities. However, NCE treatment (150 mg/kg) elevated CAT (P < 0.01), SOD (P < 0.01), and GRx (P < 0.001) activities compared to the SCZ group in the cerebral tissue.

**Table 2:** Effect of NCE treatment on cerebral antioxidant enzymes activities Values are shown as the mean ± SD of 7 mice in each group. Tukey’s Post hoc test was used to compare between groups.

Groups	CAT (U/mg protein)	SOD (% inhibition)	GRx (U/mg protein)
Control	144.82 ± 18.28	97.09 ± 1.62	132.86 ± 23.30
NCE 150	154.28 ± 15.79	94.85 ± 2.89	126.64 ± 20.69
SCZ	68.91 ± 11.89 ***	59.29 ± 6.70 ***	54.12 ± 11.62 ***
ARI	130.61 ± 30.10 ^+++^	88.71 ± 11.46 ^+++^	114.82 ± 15.27 ^+++^
KET + NCE 70	52.19 ± 20.95 ***	68.97 ± 7.73 ***	88.07 ± 19.58 ** ^+^
KET + NCE0 150	117.24 ± 21.31 ^++^	84.35 ± 13.21 ^++^	130.50 ± 19.95 ^+++^

SCZ; schizophrenia, ARI; aripiprazole, NCE (*Nostoc commune* extract) 70 and 150 mg/kg**P < 0.01, ***P < 0.001 as compared to the control group;+P<0.05, ^++^ P<0.01, ^+++^ P<0.001 as compared to SCZ group.

### 3.6 NCE treatments effects on GSH and MDA levels

Regarding Table 3, KET injection in the SCZ group caused a significant increase in cerebral MDA levels compared to the control group (p < 0/001). NCE treatment at both concentrations (70 and 150 mg/kg) significantly decreased MDA levels compared to the SCZ group (p < 0.01 and p < 0.001, respectively). On the other hand, cerebral GSH levels were reduced in the SCZ group compared to the control group (p < 0/001). NCE treatment at the 150 mg/kg dose significantly increased (P < 0.001) in the GSH levels.

**Table 3:** Effect of NCE treatment on cerebral MDA and GSH levels.Values are shown as the mean ± SD of 7 mice in each group. Tukey’s Post hoc test was used to compare between groups.

Groups	GSH (mg/gr protein)	MDA (μg/mg protein)
Control	0.33 ± 0.07	0.24 ± 0.03
NCE 150	0.31 ± 0.06	0.25 ± 0.04
SCZ	0.13 ± 0.01***	0.44 ± 0.07***
ARI	0.24 ± 0.04*^++^	0.25 ± 0.04^+++^
KET + NCE 70	0.12 ± 0.03***	0.33 ± 0.05 *^++^
KET + NCE 150	0.26 ± 0.01^+++^	0.23 ± 0.02^+++^

SCZ; schizophrenia, ARI; aripiprazole, NCE (*Nostoc commune* extract) 70 and 150 mg/kg *P < 0.05, ***P < 0.001 as compared to the control group; ^++^ P<0.01, ^+++^ P<0.001 as compared to SCZ group.

### 3.7 Effects of NCE on the expression of inflammatory factors

**Fig. 6:**
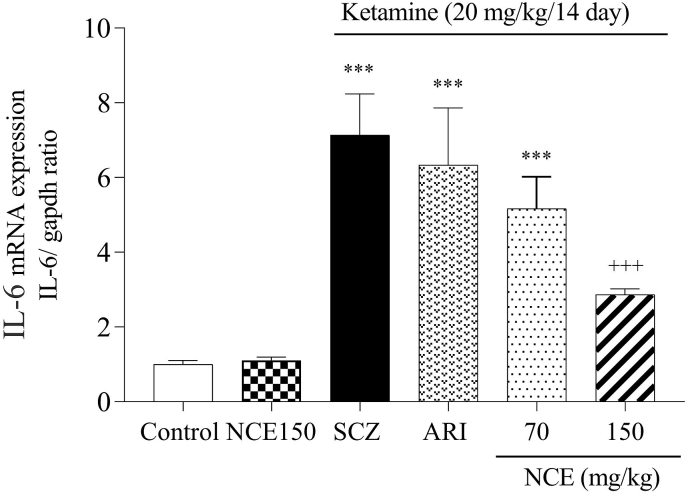
NCE treatment effects on gene expression of IL-6 in KET-induced SCZ. Values were expressed as means ± SD for 7 mice. SCZ; schizophrenia, ARI; aripiprazole, NCE (*Nostoc commune* extract) 70 and 150 mg/kg). ***P < 0.001, as compared to the control group. +++P < 0.001 as compared to the SCZ group.

**Fig. 7:**
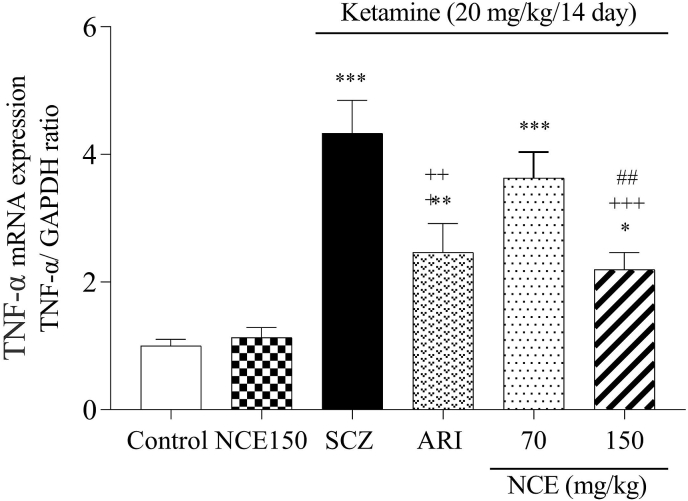
NCE treatments effects on gene expression of TNF-α in KET-induced SCZ. Values were expressed as means ± SD for 7 mice. SCZ; schizophrenia, ARI; aripiprazole, NCE (*Nostoc commune* extract) 70 and 150 mg/kg). ***P < 0.001, **P < 0.01, *P < 0.05 as compared to control group. +++P < 0.001 as compared to the SCZ group,##P < 0.01 as compared to the NCE 70 group.

As shown in Fig. 6, Fig. 7, KET injection in the SCZ group caused a significant increase in the IL-6 and TNF-α expression compared to the control group in the cortex (p < 0/001). Whereas NCE treatment at a dose of 150 mg/kg significantly decreased IL-6 and TNF-α expression compared to the SCZ group (P < 0.001, P < 0.001). Also, NCE treatment at the dose of 150 mg/kg showed a significant difference compared to 70 mg/kg of NCE in the TNF-α expression.

## 4 Discussion

According to the present study, NCE treatment improved behavioral deficits and cerebral oxidative damage induced by KET. Also, NCE reduced KET-induced neuro-inflammation, evidenced by decreased levels of inflammatory cytokines in the cortex tissue. In the current research, anxiety-like behaviors were assessed by OFT. The results showed that the SCZ group was mainly present in the surrounding area, indicating KET-induced anxiety-like behaviors. NCE treatment significantly ameliorated the anxiety-like behaviors induced by KET, as evidenced by increased presence time in the central area.

Numerous studies have shown the oxidative stress role in developing schizophrenia phenotypes, especially anxiety-like behaviors [37,38]. Oxidative stress is induced by ROS overproduction and antioxidant system defect, evidenced by the reduced GSH and antioxidant enzyme and increased lipid peroxidation. The brain tissue is highly vulnerable due to its high metabolic rate and low antioxidant level against oxidative damage [39]. In the animal model of SCZ, chronic KET exposure through the GRIN1 subunit of NMDARs up-regulation leads to increased intracellular calcium influx. As a result, calcium homeostasis imbalance causes mitochondrial dysfunction, excessive free radicals generation, and thus oxidative stress. Earlier, KET has been reported to be effective in increasing the MDA level and decreasing the GSH level and antioxidant enzyme activity in mice brains, suggesting oxidative stress contribution in SCZ [40]. There is a tight interaction between NMDAR’s function and oxidative stress. NMDAR’s blockage by KET through inhibition of the GSH system transcription gene causes reduced GSH synthesis. GSH reduction is critical in excitatory/inhibitory imbalance in GABAergic interneurons in cortex circuits, causing SCZ-like symptoms such as anxiety [37,38,41]. Consistent with previous studies, the present study indicated that chronic KET administration induced oxidative stress in the cerebral cortex, marked by reduced SOD, CAT, and GRx activities and GSH levels, and enhanced MDA levels. Treatment with NCE was able to restore cerebral antioxidant status. These results indicated that the neuroprotective effect of NCE could be related to potential antioxidant compounds such as MAAs, probably inhibiting oxidative damage via the Keap1-Nrf2-ARE pathway activation [42]. Studies have proven that *N. commune* compounds such as MAAs and polysaccharides are potent radical scavengers in vitro [17,20,43].

The hyperlocomotion in OFT could be associated with DA levels. Indeed, KET blocks NMDA receptors on GABAergic interneurons, decreasing GABA inhibitory function on dopaminergic neurons. DA release and neurotransmission elevation in the cerebral cortex promotes hyperlocomotion in rodents [44]. Hence, in the present study, DA level enhancement and hyperlocomotion after chronic administration of KET in OFT were observed, decreasing by NCE treatment. Similar to the present research results, evidence has revealed that KET induces hyperlocomotion through increased DA levels [[45], [46], [47]]. As an autoxidizable neurotransmitter, DA produces free radicals through molecular oxygen autoxidation. Cyanobacteria via antioxidant potential, as reported earlier, might prevent DA autoxidation and restore dopaminergic neurotransmission. Therefore, locomotor activity balance may be due to dopaminergic system regulation by NCE antioxidant agent [48].

On the other hand, ROS generation, followed by KET injection, activates the microglial cells, causing an increase in the release of inflammatory cytokines [12]. The enhanced microglia activation and pro-inflammatory cytokines levels are associated with neuro-inflammation in SCZ [49,50]. Neuroimaging studies have confirmed the microglia activation in patients and animal models of SCZ [51]. Also, chronic KET administration has been reported to increase IL-6 and TNF-α release, evidenced by those serum and cerebral levels elevation in mice [40,46]. In the current research, a rise in IL-6 and TNF-α levels followed by KET injection was also concomitant with the earlier studies. In contrast, KET can acts slightly as a “homeostatic regulator” of the acute and localized inflammation only in the presence of inflammatory stimulus. Also, if KET is administered before the inflammatory challenge, has inflammation-regulating effect like when KET is given for anesthesia before surgery [52]. The NCE treatment significantly decreased the overexpression of IL-6 and TNF-a in the cortex. This may be owing to the inhibition of NF-κB nuclear translocation by anti-inflammatory agents of NCE [53]. Moreover, the antioxidant system strengthened by NCE through ROS suppression can reduce microglial activation, thus preventing the overexpression of cytokines [19,20]. Previously, numerous studies have confirmed that NCE effectively reduces the expression of the inflammatory cytokines (TNFα, IL-1β, & IL-6) in mouse macrophage cell line RAW 264.7 and mouse bone marrow-derived macrophages [53]. According to Xu et al., p-Hydroxy benzaldehyde of NCE, by downregulating the mRNA and protein expression of TNF-α and IL-6, improved dextran sodium sulfate-induced colitis in mice [25]. Also, cell culture evidence has shown reduced-scytonemin derived from *Nostoc commune* by upregulating expression of Keap1/Nrf2/ARE pathway-suppressed LPS/IFN-induced inflammation [16]. According to UV absorption spectra in Fig. 2, NCE is probably rich in the MAA antioxidant compounds responsible for protective effects on oxidative and inflammatory damage in mice brains.

Evidence has focused on the TNF-α and IL-6 role in SCZ patients, showing that IL-6 reduction leads to negative symptom alleviation [54,55]. In the current study, depressive-like symptoms were analyzed by FST. By reducing antioxidant defense and increasing inflammation, chronic injection of KET caused elevated immobility duration in FST as a sign of depressive symptoms. This behavioral investigation revealed that NCE treatment remarkably ameliorated the KET-induced depressive-like behaviors, as evidenced by increased swimming time and decreased immobility time in FST. The results further confirmed the neuroprotective effects of NCE in the mice model of SCZ. These effects are likely attributed to the anti-inflammatory and antioxidant compounds of NCE.

Currently, antipsychotics, as the only treatment for SCZ, focus mainly on declining behavioral phenotypes. However, most of them are less effective in ameliorating the emotional and negative symptoms [56]. Literature has shown that adverse effect is more common among users of first-generation antipsychotics (strong D2R antagonism) such as Halopridole, which are pro-oxidant and pro-inflammatory, whereas neurotoxicity of second-generation antipsychotics (partial agonism of D2R) like ARI is often induced at high doses [57]. Also, second-generation antipsychotics have less risk of side effects such as extrapyramidal symptoms, akathisia, and dystonia than first-generation antipsychotics [56], probably owing to its intrinsic activity that does not block D2 receptors excessively [58].

Blue-green algae and microalgae that possess nutritious compounds and are safe in high dosages have indicated promising results in mood regulation and neuroprotective effects [[59], [60], [61]]. Also, in this experiment, the NCE (150 mg/kg) group without ketamine injection was applied to evaluate the side effects of the NCE. The behavioral and biochemical tests and gene expression results indicate no significant difference between this and the control groups.

## 5 Conclusion

From the behavioral and biochemical observations, it can be concluded that due to its regulating impact on dopamine, antioxidant status, and inflammation, NCE was effective against depressive and anxiety-like behaviors on KET-induced SCZ in mice. However, further studies are required to clarify the neuroprotective effects of *Nostoc commune* including direct measurement of NCE and its metabolites and protein levels of inflammatory cytokines in mice brain. We hope that resolve this limitations in the our future studies.

## Funding

This research did not receive any specific grant from funding agencies in the public, commercial, or not-for-profit sectors.

## Data availability

**T**he authors do not have permission to share data.

## CRediT authorship contribution statement

**Parisa Jahani Bahnamiri:** Project administration, Writing – original draft. **Akbar Hajizadeh Moghaddam:** Funding acquisition, Investigation, Supervision, Writing – review & editing. **Mojtaba Ranjbar:** Formal analysis, Methodology. **Ehsan Nazifi:** Methodology.

## Declaration of competing interest

The authors declare that they have no known competing financial interests or personal relationships that could have appeared to influence the work reported in this paper

## References

[1] Rodrigues-Amorim D., Rivera-Baltanas T., Spuch C., Caruncho H.J., Gonzalez-Fernandez A., Olivares J.M., Agis-Balboa R.C. (2018). Cytokines dysregulation in schizophrenia: a systematic review of psychoneuroimmune relationship. Schizophr. Res..

[2] Kanchanatawan B., Thika S., Sirivichayakul S., Carvalho A.F., Geffard M., Maes M. (2018). In schizophrenia, depression, anxiety, and physiosomatic symptoms are strongly related to psychotic symptoms and excitation, impairments in episodic memory, and increased production of neurotoxic tryptophan catabolites: a multivariate and machine learning study. Neurotox. Res..

[3] Tamminga C.A., Medoff D.R. (2022). The biology of schizophrenia. Dialogues Clin. Neurosci..

[4] McCutcheon R.A., Krystal J.H., Howes O.D. (2020). Dopamine and glutamate in schizophrenia: biology, symptoms and treatment. World Psychiatr..

[5] Chien Y.L., Hwu H.G., Hwang T.J., Hsieh M.H., Liu C.C., Lin-Shiau S.Y., Liu C.M. (2020). Clinical implications of oxidative stress in schizophrenia: acute relapse and chronic stable phase. Prog. Neuro-Psychopharmacol. Biol. Psychiatry.

[6] Kogan S., Ospina L.H., Kimhy D. (2018). Inflammation in individuals with schizophrenia–Implications for neurocognition and daily function. Brain Behav. Immun..

[7] Chen B., Zhao J., Zhang R., Zhang L., Zhang Q., Yang H., An J. (2022). Neuroprotective effects of natural compounds on neurotoxin-induced oxidative stress and cell apoptosis. Nutr. Neurosci..

[8] Ben-Azu B., Aderibigbe A.O., Ajayi A.M., Eneni A.E., Omogbiya I.A., Owoeye O., Umukoro S., Iwalewa E.O. (2019). Morin decreases cortical pyramidal neuron degeneration via inhibition of neuroinflammation in mouse model of schizophrenia. Int. Immunopharm..

[9] Dawidowski B., Górniak A., Podwalski P., Lebiecka Z., Misiak B., Samochowiec J. (2021). The role of cytokines in the pathogenesis of schizophrenia. J. Clin. Med..

[10] Fan N., Xu K., Ning Y., Rosenheck R., Wang D., Ke X., Ding Y., Sun B., Zhou C., Deng X., Tang W. (2016). Profiling the psychotic, depressive and anxiety symptoms in chronic ketamine users. Psychiatr. Res..

[11] Wang C., Liu F., Patterson T.A., Paule M.G., Slikker W. (2017). Relationship between ketamine-induced developmental neurotoxicity and NMDA receptor-mediated calcium influx in neural stem cell-derived neurons. Neurotoxicology.

[12] Réus G.Z., Simões L.R., Colpo G.D., Scaini G., Oses J.P., Generoso J.S., Prossin A.R., Kaddurah-Daouk R., Quevedo J., Barichello T. (2017). Ketamine potentiates oxidative stress and influences behavior and inflammation in response to lipolysaccharide (LPS) exposure in early life. Neurosciences.

[13] Kayser M.S., Dalmau J. (2016). Anti-NMDA receptor encephalitis, autoimmunity, and psychosis. Schizophr. Res..

[14] Kaar S.J., Natesan S., Mccutcheon R., Howes O.D. (2020). Antipsychotics: mechanisms underlying clinical response and side-effects and novel treatment approaches based on pathophysiology. Neuropharmacology.

[15] Sharifi-Rad M., Lankatillake C., Dias D.A., Docea A.O., Mahomoodally M.F., Lobine D., Chazot P.L., Kurt B., Boyunegmez Tumer T., Catarina Moreira A., Sharopov F. (2020). Impact of natural compounds on neurodegenerative disorders: from preclinical to pharmacotherapeutics. J. Clin. Med..

[16] Itoh T., Koketsu M., Yokota N., Touho S., Ando M., Tsukamasa Y. (2014). Reduced scytonemin isolated from Nostoc commune suppresses LPS/IFNγ-induced NO production in murine macrophage RAW264 cells by inducing hemeoxygenase-1 expression via the Nrf2/ARE pathway. Food Chem. Toxicol..

[17] Wang X., Yang Z., Liu Y., Wang X., Zhang H., Shang R., Laba C., Wujin C., Hao B., Wang S. (2022). Structural characteristic of polysaccharide isolated from Nostoc commune, and their potential as radical scavenging and antidiabetic activities. Sci. Rep..

[18] Wang Y., Liu J., Liu X., Zhang X., Xu Y., Leng F., Avwenagbiku M.O. (2019). Kinetic modeling of the ultrasonic-assisted extraction of polysaccharide from Nostoc commune and physicochemical properties analysis. Int. J. Biol. Macromol..

[19] Quan Y., Yang S., Wan J., Su T., Zhang J., Wang Z. (2015). Optimization for the extraction of polysaccharides from Nostoc commune and its antioxidant and antibacterial activities. J. Taiwan Inst. Chem. Eng..

[20] Nazifi E., Wada N., Asano T., Nishiuchi T., Iwamuro Y., Chinaka S., Matsugo S., Sakamoto T. (2015). Characterization of the chemical diversity of glycosylated mycosporine-like amino acids in the terrestrial cyanobacterium Nostoc commune. J. Photochem. Photobiol., B.

[21] Liao H.F., Wu T.J., Tai J.L., Chi M.C., Lin L.L. (2015). Immunomodulatory potential of the polysaccharide-rich extract from edible cyanobacterium Nostoc commune. Med. Sci..

[22] Li Z., Guo M. (2018 Mar 6). Healthy efficacy of Nostoc commune Vaucher. Oncotarget.

[23] Ku C.S., Kim B., Pham T.X., Yang Y., Weller C.L., Carr T.P., Park Y.K., Lee J.Y. (2015). Hypolipidemic effect of a blue-green alga (Nostoc commune) is attributed to its nonlipid fraction by decreasing intestinal cholesterol absorption in C57BL/6J Mice. J. Med. Food.

[24] Tseng C.C., Yeh H.Y., Liao Z.H., Hung S.W., Chen B., Lee P.T., Nan F.H., Shih W.L., Chang C.C., Lee M.C. (2021). An in vitro study shows the potential of Nostoc commune (Cyanobacteria) polysaccharides extract for wound-healing and anti-allergic use in the cosmetics industry. J. Funct.Foods.

[25] Xu X., Wei C., Yang Y., Liu M., Luo A., Song H., Wang Y., Duan X. (2021). New discovery of anti-ulcerative colitis active ingredients of Nostoc commune: p-Hydroxy benzaldehyde. J. Funct.Foods.

[26] Tsai S.C., Huang Y.W., Wu C.C., Wang J.J., Chen Y.T., Singhania R.R., Chen C.W., Dong C.D., Hsieh S.L. (2022). Anti-obesity effect of Nostoc commune ethanol extract in vitro and in vivo. Nutrients.

[27] Guo M., Ding G.B., Guo S., Li Z., Zhao L., Li K., Guo X. (2015). Isolation and antitumor efficacy evaluation of a polysaccharide from Nostoc commune Vauch. Food Funct..

[28] Kraeuter A.K., Guest P.C., Sarnyai Z. (2019).

[29] Dalvi A., Lucki I. (1999). Murine models of depression. Psychopharmacol..

[30] Guo L., Zhang Y., Li Q. (2009). Spectrophotometric determination of dopamine hydrochloride in pharmaceutical, banana, urine and serum samples by potassium ferricyanide-Fe (III). Anal. Sci..

[31] Genet S., Kale R.K., Baquer N.Z. (2002). Alterations in antioxidant enzymes and oxidative damage in experimental diabetic rat tissues: effect of vanadate and fenugreek (Trigonella foenum graecum). Mol. Cell. Biochem..

[32] Pinto R.E., Bartley W. (1969). The effect of age and sex on glutathione reductase and glutathione peroxidase activities and on aerobic glutathione oxidation in rat liver homogenates. Biochem. J..

[33] Fukuzawa K., Tokumurai A. (1976). Glutathione peroxidase activity in tissues of vitamin E-deficient mice. J. Nutr. Sci. Vitaminol..

[34] Esterbauer H., Cheeseman K.H. (1990).

[35] Bradford M.M. (1976). A rapid and sensitive method for the quantitation of microgram quantities of protein utilizing the principle of protein-dye binding. Anal. Biochem..

[36] Moghaddam A.H., Sangdehi S.R., Ranjbar M., Hasantabar V. (2020). Preventive effect of silymarin-loaded chitosan nanoparticles against global cerebral ischemia/reperfusion injury in rats. Eur. J. Pharmacol..

[37] Krolow R., Arcego D.M., Noschang C., Weis S.N., Dalmaz C. (2014). Oxidative imbalance and anxiety disorders. Curr. Neuropharmacol..

[38] Perkins D.O., Jeffries C.D., Do K.Q. (2020). Potential roles of redox dysregulation in the development of schizophrenia. Biol. Psychiatr..

[39] Rossetti A.C., Paladini M.S., Riva M.A., Molteni R. (2020). Oxidation-reduction mechanisms in psychiatric disorders: a novel target for pharmacological intervention. Pharmacol. Ther..

[40] Yadav M., Parle M., Jindal D.K., Dhingra S. (2018). Protective effects of stigmasterol against ketamine-induced psychotic symptoms: possible behavioral, biochemical and histopathological changes in mice. Pharmacol. Rep..

[41] Bouayed J., Rammal H., Soulimani R. (2009). Oxidative stress and anxiety: relationship and cellular pathways. Oxid. Med. Cell. Longev..

[42] Gacesa R., Lawrence K.P., Georgakopoulos N.D., Yabe K., Dunlap W.C., Barlow D.J., Wells G., Young A.R., Long P.F. (2018). The mycosporine-like amino acids porphyra-334 and shinorine are antioxidants and direct antagonists of Keap1-Nrf2 binding. Biochimie.

[43] Wada N., Sakamoto T., Matsugo S. (2015). Mycosporine-like amino acids and their derivatives as natural antioxidants. Antioxidants.

[44] Sun L.H., Fan Y.Y., Wang X., Zheng H.B. (2020). Pharmacodynamic elucidation of glutamate & dopamine in ketamine-induced anaesthesia. Chem. Biol. Interact..

[45] Yadav M., Jindal D.K., Dhingra M.S., Kumar A., Parle M., Dhingra S. (2018). Protective effect of gallic acid in experimental model of ketamine-induced psychosis: possible behaviour, biochemical, neurochemical and cellular alterations. Inflammopharmacology.

[46] Yadav M., Jindal D.K., Parle M., Kumar A., Dhingra S. (2019). Targeting oxidative stress, acetylcholinesterase, proinflammatory cytokine, dopamine and GABA by eucalyptus oil (Eucalyptus globulus) to alleviate ketamine-induced psychosis in rats. Inflammopharmacology.

[47] Yadav M., Parle M., Jindal D.K., Sharma N. (2018). Potential effect of spermidine on GABA, dopamine, acetylcholinesterase, oxidative stress and proinflammatory cytokines to diminish ketamine-induced psychotic symptoms in rats. Biomed. Pharmacother..

[48] Haider S., Shahzad S., Batool Z., Sadir S., Liaquat L., Tabassum S., Perveen T. (2021). Spirulina platensis reduces the schizophrenic-like symptoms in rat model by restoring altered APO-E and RTN-4 protein expression in prefrontal cortex. Life Sci..

[49] Na K.S., Jung H.Y., Kim Y.K. (2014). The role of pro-inflammatory cytokines in the neuroinflammation and neurogenesis of schizophrenia. Prog. Neuro-Psychopharmacol. Biol. Psychiatry.

[50] Meyer U., Schwarz M.J., Müller N. (2011). Inflammatory processes in schizophrenia: a promising neuroimmunological target for the treatment of negative/cognitive symptoms and beyond. Pharmacol. Ther..

[51] Müller N. (2018). Inflammation in schizophrenia: pathogenetic aspects and therapeutic considerations. Schizophr. Bull..

[52] De Kock M., Loix S., Lavand’homme P. (2013). Ketamine and peripheral inflammation. CNS Neurosci. Ther..

[53] Ku C.S., Pham T.X., Park Y., Kim B., Shin M.S., Kang I., Lee J. (2013). Edible blue-green algae reduce the production of pro-inflammatory cytokines by inhibiting NF-κB pathway in macrophages and splenocytes. Biochim. Biophys. Acta, Gen. Subj..

[54] Al-Hakeim H.K., Al-Rammahi D.A., Al-Dujaili A.H. (2015). IL-6, IL-18, sIL-2R, and TNFα proinflammatory markers in depression and schizophrenia patients who are free of overt inflammation. J. Affect. Disord..

[55] Zhang L., Zheng H., Wu R., Zhu F., Kosten T.R., Zhang X.Y., Zhao J. (2018). Minocycline adjunctive treatment to risperidone for negative symptoms in schizophrenia: association with pro-inflammatory cytokine levels. Prog. Neuro Psychopharmacol. Biol. Psychiatr..

[56] Stępnicki P., Kondej M., Kaczor A.A. (2018). Current concepts and treatments of schizophrenia. Molecules.

[57] De Simone G., Mazza B., Vellucci L., Barone A., Ciccarelli M., de Bartolomeis A. (2023). Schizophrenia synaptic pathology and antipsychotic treatment in the framework of oxidative and mitochondrial dysfunction: translational highlights for the clinics and treatment. Antioxidants.

[58] Kikuchi T., Maeda K., Suzuki M., Hirose T., Futamura T., McQuade R.D. (2021). Discovery research and development history of the dopamine D2 receptor partial agonists, aripiprazole and brexpiprazole. Neuropsychopharmacol. Rep..

[59] Mutoti M., Gumbo J., Jideani A.I. (2022). Occurrence of cyanobacteria in water used for food production: a review. Phys. Chem. Earth, Parts A/B/C.

[60] McCarthy B., O’Neill G., Abu-Ghannam N. (2022). Potential psychoactive effects of microalgal bioactive compounds for the case of sleep and mood regulation: opportunities and challenges. Mar. Drugs.

[61] Ramos V., Reis M., Ferreira L., Silva A.M., Ferraz R., Vieira M., Vasconcelos V., Martins R. (2023). Stalling the course of neurodegenerative diseases: could cyanobacteria constitute a new approach toward therapy?. Biomolecules.

